# Assessment of prescription pattern at the public health facilities of Lucknow district

**DOI:** 10.4103/0253-7613.45148

**Published:** 2008

**Authors:** Ranjeeta Kumari, M.Z. Idris, Vidya Bhushan, Anish Khanna, Monika Agrawal, Shivendra Kumar Singh

**Affiliations:** Department of Community Medicine, King George Medical University, Lucknow, Uttar Pradesh, India

**Keywords:** Cost, multi-stage stratified sampling, prescription pattern

## Abstract

**Objectives:**

To study the prescription pattern at the different levels of public health facilities of Lucknow district and to assess the average cost of drugs prescribed.

**Methods:**

Multi-stage stratified random sampling was done to select 1625 prescriptions of the patients attending the different level of public health facilities in Lucknow district, from August 2005 to September 2006, which was used for the development of study tools, collection of data and analysis.

**Results:**

The important components of prescription viz. examination findings, weight of the child, follow up visit and the signatures of the prescribers were absent in the prescriptions at the primary level. Polypharmacy was common (3.1 ± 1.6 drugs per prescription). The prescription of drugs by generic name was low (27.1%). The prescriptions at the secondary level health facilities were incomplete with respect to mentioning the suffix/prefix of the drug, full name, dose, frequency and strength of the drugs, and directions specifying the route and duration of the treatment. The average cost of drugs/prescription/day in US$ (Mean, SD) was found to be the highest at the tertiary level (0.34, 0.43), which decreased significantly at the primary level health facilities.

**Conclusion:**

The pattern of prescription in terms of completeness and rationality was poor. There is an urgent need to improve the standards of drug prescription.

## Introduction

Drugs play an important role in protecting, maintaining and restoring health. Prescription writing is a science and an art, as it conveys the message from the prescriber to the patient. The treatment of diseases by the use of essential drugs, prescribed by their generic names, has been emphasized by the WHO and the National Health Policy of India.[1] The International Network for the Rational Use of Drugs (INRUD) generated indicators in three main drug use areas viz prescribing, patient care and drug systems.[2]

The cost of drug prescription poses problems in developing countries such as India, which allocates only 0.9% of its Gross Domestic Product (GDP), i.e. Rs. 200 per capita,[3] to health. The allocation for meeting the cost of the drugs is even meager. Moreover, the production of pharmaceutical preparations in India is grossly imbalanced and there is cut throat competition among drug companies, which breeds malpractice. Indian markets are flooded with over 70,000 formulations, as compared to about 350 listed in the WHO essential drug list, and pharmaceutical companies encourage doctors to prescribe branded medicines, often in exchange for favors. This study was, therefore, undertaken with the aim to find out the prescription pattern and cost per prescription at different levels of health facilities in the public health facilities of Lucknow - the capital city of Uttar Pradesh, a state in north India.

## Materials and Methods

A cross-sectional study was conducted from August 2005 to September 2006, among patients attending the allopathic public health facilities of Lucknow district, which provide three different levels of health care. The sample size was 1688 (*P* = 50% for the level of completeness of prescription, d = 5%, which is the absolute permissible error in the prevalence, with a 95% confidence limit; nonresponse/wastage = 10%). We were able to obtain the required information from 1625 patients only. Multi-stage stratified random sampling was used to select the public health facilities to be included in the study. At the first stage (tertiary level), Medical College (MC) was selected. At the second stage (secondary level), the Balrampur District Hospital (DH) and two community health centers (CHC) were selected. At the third stage (primary level), two primary health centers (PHC) each, under the two selected CHCs were selected. Based on the previous year's out patient department (OPD) attendance, the number of patients to be included in the study at each of these health facilities was estimated by probability proportion to size (PPS).

**Figure 1:**
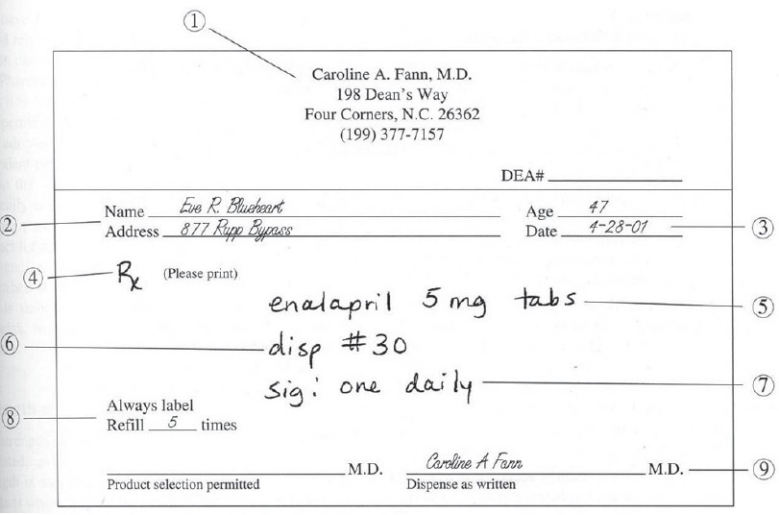
The ideal prescription as in the Principles of Prescription Order Writing by Laurela Edwards and Dan M. Roden in Goodman and Gilman's, The Pharmacological Basis of Therapeutics, Tenth edition

At the MC, 844 new patients were interviewed from the OPDs of 10 departments i.e. Medicine, General Surgery, Obstetrics and Gynecology, Pediatrics, Orthopedics, Otorhinolaryngology, Ophthalmology, Cardiology, Neurology, and Tuberculosis and Chest diseases. At the DH, a total of 420 new patients attending the OPDs of Medicine, General Surgery, Obstetrics and Gynecology, and Pediatrics were interviewed. One hundred and five new patients were interviewed from each of these departments. Similarly, 105 new patients were interviewed from each of the two randomly selected CHCs, while 53 patients from each of the selected PHCs were interviewed. To assess the completeness of prescription, the variables were taken according to the Principles of Prescription Order Writing[4] [Figure 1]. The patients attending the OPDs of the respective health care facility were selected by systematic random sampling for the interview. The calculation of cost of the prescription included the cost of drugs only.

‘New’ (any patient attending the health facility for the first time for the present episode of illness) or ‘referred’ (a patient who had visited some other health facility for the present episode of illness and was referred to the present health care facility) patients attending the OPD of the respective health care facility were included in the study, while patients whose family members worked in the health care facility and follow-up patients were excluded.

Permission to conduct the study was taken from the superintendents of the respective health care facility. All the patients were interviewed after taking informed verbal consent from them. The prescription was photographed by a digital camera after they had consulted the doctor. The prescribing doctor was kept unaware of the procedure, except in unavoidable circumstances. In the case of pediatric patients, the adult accompanying was the respondent. A quantitative pretested structured interview schedule was used to record information.

### Statistical analysis

Data was analysed using the software Epi Info version 6[5] and Microsoft Excel (Analysis toolpak) for Windows. Discrete data was analysed using Pearson's Chi-square test for normal distribution and Single factor anova for the comparison of means. *P* value < 0.05 was considered significant.

## Results

Observation of the prescriptions revealed that patient details such as age, gender and address were lacking in considerable prescriptions, specially so at the DH. The prescriptions at the PHCs and CHCs lacked all details about the prescriber. The data from other health facilities were also not very impressive.

The details of the chief complaints of the patients and the legibility of the prescriptions were significantly better in the prescriptions at the tertiary and secondary levels, as compared to the primary health facilities. The details of examination findings, weight of the child, follow up visit and signature of the prescriber were absent in the prescriptions at the PHC [Table 1].

**Table 1:** Assessment of the correctness of components of prescription in the public health facilities

*Variables*	*Level of care*									
	
	*Tertiary*		*Secondary*				*Primary*		*Total (1625)*	
				
	*MC (817)*		*DH (401)*		*CHC (202)*		*PHC (205)*		
	*N*	%	*N*	%	*N*	%	*N*	%	*N*	%
Date	293	35.8	401	100	101	50	205	100	1000	61.5
*P* value	0.000*		0.000*		0.000*			
Chief complaints	430	52.6	118	29.4	68	33.6	28	13.6	644	39.6
*P* value	0.059		0.000*		0.000*			
Examination findings	333	40.7	36	9	14	6.9	0	0	383	23.6
*P* value	0.000*		0.000*		0.000*			
Weight of the child	60/ 121	49.6	1/119	0.8	0/ 57	0	0/ 70	0	61/ 428	14.2
*P* value	0.000*		1.000		0.000*			
Provisional diagnosis	435	53.2	94	23.4	22	10.9	45	21.9	596	36.7
*P* value	0.000*		0.45		0.000*			
Follow-up visit mentioned	197	24.1	8	2.0	1	0.5	0	0	206	12.7
*P* value	0.000*		0.071		0.000*			
Date and Day	34	17.2	6	75	1	100	0	0	41	19.9
*P* value	-		-						
Legibility	778	95.2	368	91.7	75	37.1	106	51.7	1327	81.6
*P* value	0.000*		0.000*		0.000*			
Signature with last name written in full	134	16.4	14	3.5	2	1.0	0	0	150	9.2
*P* value	0.000*		0.016*		0.000*			
Duration of treatment	1329	61.6	154	12.0	45	5.1	26	3.5	1554	30.8
*P* value	0.000*		0.000*		0.000*			
Directions specifying the route	552	25.6	9	0.7	7	0.8	33	4.5	601	11.9
*P* value	0.000*		0.000*		0.000*			
Non-pharmacological treatment^§^	158/817	19.3	11/ 401	2.7	2/202	0.9	4/ 205	1.9	175/1625	10.7
*P* value	0.000*		1.0		0.000*			
Language used					
Hindi	228	10.6	6	0.5	5	0.5	0	0	239	4.7
English	219	10.1	12	0.9	5	0.5	3	4.0	239	4.7
Latin	1702	78.9	892	69.3	205	23.6	172	23.4	2971	58.8
Symbol	1323	61.3	201	15.6	495	56.9	161	21.9	2180	43.2

* Significant *P* values <0.05 calculated by Chi Square test for Proportion. *P* values in the first column, second column and third column depict the association of variables between Tertiary and Secondary level, Secondary and Primary level and Tertiary and Primary level respectively.

Prescription of the drugs by generic names was disheartening at the tertiary level (1.1%), while the use of abbreviations in writing the name of the drugs was the maximum at the primary level (69.8%). The details about the dose, strength, frequency and duration of treatment, and non-pharmacological treatment were best at the tertiary level [Table 2].

**Table 2:** Assessment of the pattern of prescription in the public health facilities

*Details of drugs*	*Level of care*												
	
	*Tertiary*			*Secondary*						*Primary*		*Total (5048)*	
				
	*MC (2157)*			*DH (1287)*			*CHC (869)*			*PHC (735)*			
						
	n	%	n	%	n	%	n	%	n	%			
Generic name	24		1.1	724		56.2	557		64.1	571	77.7	1876	27.1
*P* value	0.000*			0.000*			0.000*						
Full name	2146		99.4	748		58.1	401		46.1	222	30.2	3517	69.6
*P* value	0.000*			0.000*			0.000*						
Suffix/prefix	1887		87.4	429		33.3	347		39.9	246	33.4	2909	57.6
*P* value	0.000*			0.2			0.000*						
Dose§	1795/2087		86.0	912/1254		72.7	381/845		45.1	302/706	42.7	3390/4892	69.3
*P* value	0.000*			0.000*			0.000*						
Dose for liquids as Tsf§	210/ 271		77.4	111/137		81.0	21/ 92		22.8	21/44	47.7	363/ 544	66.7
*P* value	0.000*			0.29			0.000*						
Strength of the drug	730		33.8	69		5.3	37		4.2	13	1.7	849	16.8
*P* value	0.000*			0.016*			0.000*						
Frequency of the drug	2037		94.4	987		76.7	661		76.0	313	42.6	3998	79.1
*P* value	0.000*			0.000*			0.000*						

* Significant *P* values <0.05 calculated by Chi Square test for Proportion. *P* values in the first column, second column and third column depict the association of variables between Tertiary and Secondary level, Secondary and Primary level and Tertiary and Primary level respectively.

Polypharmacy (>2 drugs) was evident in a majority of the prescriptions, at all the health facilities. The average number (Mean ± SD) of drugs prescribed was minimum (2.6 ± 1.6) at the tertiary level, while it increased at the primary level (3.5 ± 1.4) [Table 3]. It was observed that vitamins and other supplements (25.6%) constituted the major group of drugs prescribed at all the health facilities, followed by antibiotics and antiinfectives (20.6%), and non steridal antiinflammatory drugs (NSAIDS) /antipyretics (17.7%). All the patients at the primary health care level were prescribed a drug indicating the placebo prescription at the primary level.

**Table 3:** Incidence of polypharmacy at different levels of health care facilities

*No. of drugs per prescription*	*Number of prescriptions at different levels of care*									
	
	*Tertiary*		*Secondary*				*Primary*		*Total (1625)*	
				
	*Medical college (817)*		*DH (401)*		*CHC (202)*		*PHC (205)*		*n*	*%*
						
	*n*	*%*	*n*	*%*	*n*	*%*	*n*	*%*		
0	75	9.2	14	3.5	3	1.5	0	0	92	5.7
1	126	15.4	17	4.2	7	3.5	4	2.0	154	9.5
2	203	24.8	78	19.5	13	6.4	28	13.7	322	19.8
3	172	21.1	146	36.4	48	23.8	95	46.3	461	28.4
4	155	19.0	90	22.4	45	22.3	37	18.0	327	20.1
>5	86	10.5	56	13.9	86	42.6	41	20	269	16.6
Range	0-9		0-10		0-9		1-9		0-10	
(Mean ± 2SD)	2.6 ± 2(1.6)		3.2 ± 2(1.4)		4.3 ± 2(1.7)		3.5 ± 2(1.4)		3.1 ± 2(1.6)	

*P* values <0.05 are significant, X^2^ =289.2, d.f.=15, *P*<0.0001

**Figure 2:**
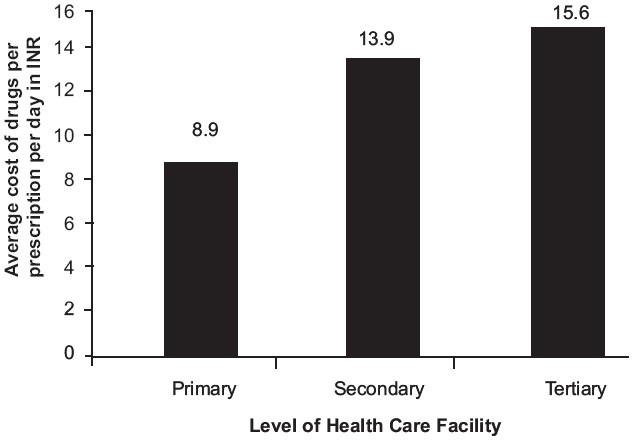
Assessment of cost of prescription, according to the level of the health care facility

The average cost of drugs/prescription/day (Mean, SD) in US$[6] (as on 29 Sep. 2006) was found to be significantly more at the MC (0.34, 0.42) and CHC (0.34, 0.26), while it was the least at the PHC (0.19, 0.19) [Figure 2].

The prescription of antibiotics and anti-infectives, NSAIDS and antipyretics and vitamins and other supplements was more at the secondary and primary levels, as compared to the tertiary level. On the other hand, a significantly greater number of H_2_ receptor antagonist/other gastrointestinal (GI) drugs, antihypertensives/other cardiovascular (CV) drugs, central nervous system (CNS) drugs, topical nasopharyngeal/ocular drugs and sedatives/anxiolytics were prescribed at the tertiary level, as compared to the secondary and primary level. Prescription of antihistamines was more at the primary level (11.1%), than at the other levels.

## Discussion

This study was an attempt to find the existing pattern of prescription writing in various public health facilities, which cater to the health needs of the majority of the less educated population. The relative lack of information about the patient and the prescriber, reported in this study, was similar to that reported by Mallet *et al*.[7] The absence of examination findings on the prescription would lead to problems in tracing the progress during the follow up visits. Also, lack of information on the weight of the child on the prescription and poor legibility could lead to medication errors during dispensing.

Polypharmacy, at all the health facilities provides a fertile ground for drug interactions. The only consolation was the practice at the tertiary level, with the lowest average (2.6 ± 1.6), along with a considerable proportion of prescriptions without any drugs. A similar trend of polypharmacy was reported by others.[8–12] Low (27.1%) generic prescription of the drugs, especially at the tertiary level health facility (1.1%) could reflect the dominating influence of pharmaceutical companies. Our findings regarding generic prescribing are contrary to those of several studies carried out in other countries,[13–16] while being similar to that of various studies carried out in India and the neighboring countries.[7,10–12,17–20]

Prescriptions written at the tertiary level, which is a teaching and training institution with its expert prescribers as well as a large group of resident doctors, were better in respect to the details of the drugs. On the other hand, the prescriptions at primary level health facilities, and to some extent the secondary level health facilities, were poor, with most of the details missing. Similar findings regarding the incompleteness of the prescription were observed by Mallet *et al.*,[7] Moghadamnia *et al.*,[16] Dineshkumar *et al*.[21] and Patel *et al*.[22] The use of Latin in writing the directions regarding the drugs could lead to administration of the wrong doses of the drugs, due to misinterpretation.

Our observation regarding the dominance of vitamins and other supplements, antibiotics and NSAIDS and antipyretics in the prescriptions at the secondary and primary level health facilities was similar to that of other studies.[8–10,16,18,21,23] The use of injections (1.7% of all the drugs) at all the health facilities in our study was similar to that observed by Rehan *et al*.[24] (0.9%), but lower than those reported in other studies.[10,22,25] Prescription of a significantly greater number of hormones, particularly steroids, at the primary level health facilities, indicates irrational prescribing.

The prescription of a greater number of H_2_ receptor antagonist/other GI drugs (11.5%), antihypertensives/other CV drugs (7.3%), CNS drugs (4.5%), topical nasopharyngeal/ocular drugs (10.7%) and sedatives/anxiolytics (4%) at the tertiary level is in accordance with the diagnosis.

The high average cost of the drugs at the MC was due to the type and severity of the illness that the patients come with. On the other hand, the high cost of the drugs at the CHCs indicate a significant degree of irrational prescribing, as is evident by the fact that it has the highest average number of drugs prescribed. We did not evaluate the cost of other aspects of health care such as transport, investigations, stay in the hospital and other intangible costs, which, if calculated, will provide us with a more realistic picture of the existing situation. We, therefore, need to devise mechanisms to keep a check on the irrational prescription of drugs.

## Conclusion

Our study reveals that despite all the efforts taken by the government and the WHO, the pattern of prescription in terms of completeness and rationality remains poor. There is an urgent need to develop standards of drug prescription and develop ways and means to ensure that they are adhered to. Special attention needs to be given to the primary and secondary level health facilities, where significant irrational prescribing in terms of polypharmacy and relative absence of the directions about the use of drugs was evident. This could be done by making it mandatory for the prescribers to attend regular continuing medical education (CME), so as to update their knowledge. A check on the influence of pharmaceutical companies and their representatives needs to be maintained in health institutions, to minimize their influence on the drug prescription. All these measures would go a long way in providing optimal, low cost, and effective medicines to the patients.
